# Systematic Genetic Analysis Identifies Cis-eQTL Target Genes Associated with Glioblastoma Patient Survival

**DOI:** 10.1371/journal.pone.0105393

**Published:** 2014-08-18

**Authors:** Qing-Rong Chen, Ying Hu, Chunhua Yan, Kenneth Buetow, Daoud Meerzaman

**Affiliations:** Center for Biomedical Informatics and Information Technology, National Cancer Institute, National Institutes of Health, Bethesda, Maryland, United States of America; University of Otago, New Zealand

## Abstract

Prior expression quantitative trait locus (eQTL) studies have demonstrated heritable variation determining differences in gene expression. The majority of eQTL studies were based on cell lines and normal tissues. We performed cis-eQTL analysis using glioblastoma multiforme (GBM) data sets obtained from The Cancer Genome Atlas (TCGA) to systematically investigate germline variation’s contribution to tumor gene expression levels. We identified 985 significant cis-eQTL associations (FDR<0.05) mapped to 978 SNP loci and 159 unique genes. Approximately 57% of these eQTLs have been previously linked to the gene expression in cell lines and normal tissues; 43% of these share cis associations known to be associated with functional annotations. About 25% of these cis-eQTL associations are also common to those identified in Breast Cancer from a recent study. Further investigation of the relationship between gene expression and patient clinical information identified 13 eQTL genes whose expression level significantly correlates with GBM patient survival (p<0.05). Most of these genes are also differentially expressed in tumor samples and organ-specific controls (p<0.05). Our results demonstrated a significant relationship of germline variation with gene expression levels in GBM. The identification of eQTLs-based expression associated survival might be important to the understanding of genetic contribution to GBM cancer prognosis.

## Introduction

Gene expression levels can be considered as quantitative traits and genetic polymophisms associated with transcript levels are referred as expression quantitative trait loci (eQTL). Substantial eQTL mapping studies have detected significant levels of polymorphism controlling individual genes, indicating that germline variations can affect gene expression networks and gene expression levels are heritable [1]–[3]. Most of these global eQTL analyses have been conducted in cell lines and normal tissues. Genome-wide association studies (GWAS) in cancer have identified a significant number of cancer susceptibility regions associated with specific cancers (http://www.genome.gov/gwastudies/). Trait-associated single nucleotide polymorphisms (SNPs) from GWAS are enriched for eQTLs for many phenotypes [4]. While several studies have combined GWAS findings and eQTL analysis to evaluate the effect of the trait-associated risk polymorphisms on transcript abundance in tumors [5]–[7], some eQTL studies have also investigated global germline impact on gene expression in tumors [5]–[9]. A systematic analysis of germline influence on gene expression tumors could identify novel alleles that influence tumorigenesis but are undetectable by analysis of normal tissue [8].

Glioblastoma multiforme (GBM) remains to be the most common and lethal primary brain tumor despite improvements in clinical care over the last 20 years. It is important to understand the inherited genetic contribution to tumor gene expression to gain insight into the underlying biology for this rapidly fatal disease. Previous studies have looked at the somatic variations and gene expression patterns observed in tumors to identify possible causal genes and pathways in GBM [10]–[11]. In the work described below we examine the role of global, inherited variation by performing cis-eQTL analysis using GBM data sets obtained from The Cancer Genome Atlas (TCGA) to systematically investigate germline contribution to tumor gene expression.

## Materials and Methods

### Data sets

GBM patient germline genotype data were obtained from blood, tumor gene expression data, organ-specific control gene expression data and clinical information were downloaded from The Cancer Genome Atlas (TCGA) in June, 2011 (http://cancergenome.nih.gov/).

### Genotype SNP6 data

Germline genotype data was obtained for 428 GBM patients with genotype calls for 906,600 SNP probes that were assayed using the Affymetrix GenomeWide SNP6.0 platform and processed using Birdseed. Genotype calls were coded as 0, 1 or 2 and filtered according to a Birdseed confidence threshold of 0.05; the genotype with more than 25% low confidence calls was excluded. Allele frequencies for each SNP were computed and SNPs with minor allele frequencies <0.1 were excluded from further analysis; as were SNPs out of Hardy Weinberg equilibrium with p<10^−4^. A total of 590,746 SNPs passed the filtering.

### Gene expression data

Level 3 tumor and organ-specific control gene expression data from Affymetrix HT HG-U133A array platform contains expression levels for 12042 genes from 287 tumor samples corresponding to individuals with SNP6 germline genotype data. To eliminate genes with low variability across patients, we kept genes with a median absolute deviation (MAD) higher than 0.5, resulting in a total of 4555 genes.

### Association analysis

cis-eQTL analysis was carried out for the 287 individuals with both SNP and gene expression data using linear regression method. We evaluated associations between the genotype and gene expression in cis by testing all SNPs within a 1 Mb window of up and downstream of the transcription start site (TSS) of a given gene. Transcripts and SNPs located on chromosomes X and Y were excluded from the study, resulting in a total of 4359 genes and 489,087 SNPs with 1,736,706 cis associations. P values were adjusted using the Benjamini Hochberg method for multiple testing corrections and a significant association was defined as False Discovery Rate (FDR)<0.05. The analysis flowchart is presented in Figure S1.

### Functional analysis of significant eQTL genes

Genes that are significantly associated with their cis-eQTLs were submitted to David Bioinformatics Resources 6.7 (http://david.abcc.ncifcrf.gov/) for disease enrichment analysis using the information from the Genetic Association Database (GENETIC_ASSOCIATION_DB_DISEASE). FDR<0.05 was set as the cut-off criteria.

### Functional annotation of significant cis-eQTLs

The significant cis-eQTLs were annotated using RegulomeDB [12] by searching for overlapping of eQTLs in the database, where the scoring system represents with increasing confidence that a variant lies in a functional location and likely results in a functional consequence. The variants that are known eQTLs for genes, and thus have been shown to be associated with expression, as most likely to be significant are labeled Category 1; those eQTLs not associated with any other functional annotation are labeled Category 6 [12].

### Comparison of the significant cis-eQTL associations with the published results

The cis-eQTL associations identified from two published studies^7, 9^ were used to perform comparison. For the Breast Cancer study^7^, the cis-eQTL association results of both ER-positive and ER-negative Breast Cancer were used. For the GBM study^9^, the cis association results from both adjusted and unadjusted analyses were obtained and their affymetrix probe sets were matched to HUGO symbol for comparison using Affymetrix annotation file.

### Survival analysis

The probability of survival and significance was calculated using the Kaplan-Meier method and logrank test. For each given gene, Cox proportional hazard modeling was used to determine the hazard ratio and confidence interval for patient overall survival stratified by high and low gene expression. Expression values of a gene were dichotomized into high and low expression using the median as a cutoff for Kaplan-Meier analysis.

## Results

### Identification of cis-eQTLs in GBM samples

A total of 287 TCGA GBM patient samples had both Affymetrix expression data from tumor samples and germline genotype data from the matched blood samples. After data pre-processing and filtering, 4359 genes and 489,087 SNP loci within 1 Mb on either side of gene transcription start site remained for cis-eQTL analysis. Association between the genotype of SNP locus and gene expression was evaluated using linear regression analysis for 1,736,706 unique SNP and gene pairs. A total of 985 significant associations were identified after adjusting (FDR<0.05) with P value< = 2.835e-05 (Table S1). These significant associations mapped to 978 SNP loci and 159 unique cis-genes. Genes and their significant associated cis-eQTLs are listed in Table S2. Of the target genes, 61 (38.4%) are associated with one SNP locus and 98 (62.6%) associated with multiple SNP loci ranging from 2 to 64. Of the 978 cis-eQTLs, 971 (99.3%) are associated with one target gene and only 7 loci associated with 2 genes.

### Neurological disease and Brain cancer genes enriched in target genes

Enrichment analysis using DAVID Bioinformatics resources (http://david.abcc.ncifcrf.gov/) showed that gene sets for multiple diseases in GENETIC_ASSOCIATION_DB_DISEASE category including neurological disease and brain cancer are enriched in the target genes (FDR<0.05, Table S3). The analysis used the information from the Genetic Association Database (http://geneticassociationdb.nih.gov), which is an archive of human genetic association studies of complex diseases and disorders. Genetic variations at a set of sixteen target genes (*C2ORF3*, *CAT*, *CRYZ*, *DARC*, *GSTM3*, *GSTT1*, *HLA-DQA1*, *HLA-DQB1*, *HPR*, *IL4R*, *LOX*, *MRPL19*, *MTR*, *NQO2 PRPH*, and *PSMB8*) were suggested to be associated with neurological disease; and polymorphisms of five target genes (*GSTM3*, *GSTT1*, *MGMT*, *MTR*, and *IL4R*) were suggested as risk factors for brain cancer. The gene sets for several non-Neurolgocal or non-GBM diseases are also enriched with many of genes sharing with Neurological or GBM diseases (Table S3). Using *GSTT1* as an example, there were 28 cis-acting SNPs linked to the expression of *GSTT1* in GBM samples (Table S1 & Table S2), which were viewed in the eQTL plot around the region of these *GSTT1* cis-acting SNPs (Figure S2); many of these significant cis-acting SNPs appear in a tight linkage disequilibrium (LD) block (Figure S2).

### Cis-eQTL associations shared between GBM samples and normal tissues/cell lines

**Figure 1:**
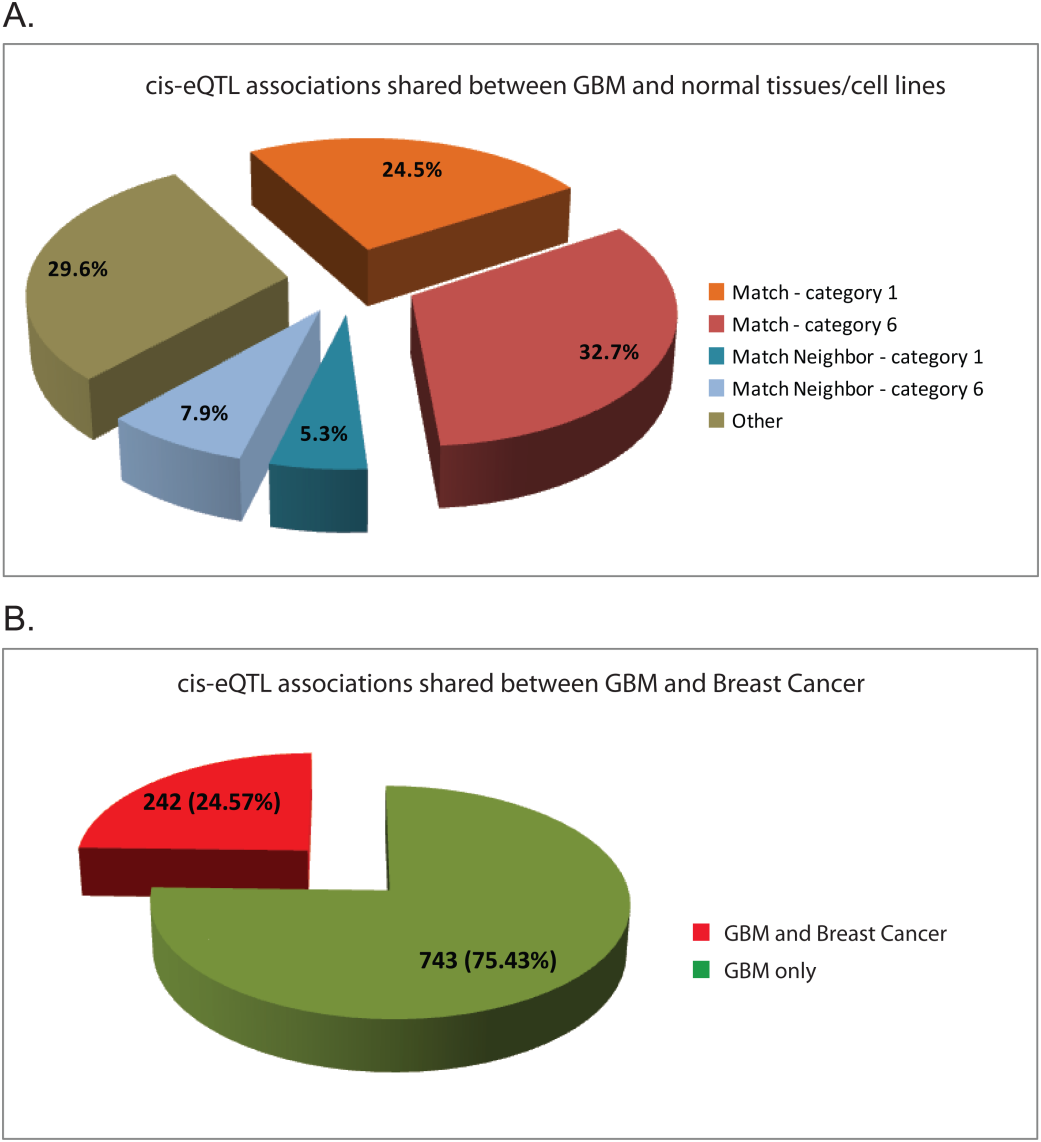
Sharing of cis-eQTL associations among different studies. **A.** cis-eQTL associations shared between GBM tumors and normal tissues/cell lines in RegulomeDB. Of 985 significant transcript-genotype associations in GBM, 57.1% (24.5%+32.7%) shared the same cis-eQTL/gene pair with cell lines or normal tissues (“Match”); 13.2% (5.3%+7.9%) shared the same cis-eQTLs which are associated with the gene in the neighbor of the target gene identified from GBM (“Match Neighbor”). ReguomeDB variant classification: category 1- known eQTLs for genes; category 6–unknown. **B.** cis-eQTL associations shared between GBM tumors identified from this study and breast cancer samples identified from a published study. Of 985 significant transcript-genotype associations in GBM, 242 (24.57%) shared the same cis-eQTL associations with breast cancer samples.

RegulomeDB is a database that annotates SNPs with known and predicted regulatory elements in the intergenic regions of the human genome [12]. It includes a large collection of eQTLs covering 142,945 SNPs derived from 9 publications. Using this database, we examined the overlaps between GBM cis-eQTLs identified from this study and the ones reported previously from normal tissues and cell lines. Of 985 significant cis-eQTL associations in GBM, a total of 563 (57.1%) shared the same cis-eQTL and gene pair with cell lines or normal tissues (Figure 1A); 241 (42.7%) of these shared associations were classified as Category 1 and known to be associated with functional annotations, suggesting that they are likely to be significant and affect binding to the target gene [12]. Furthermore, 130 (13.2%) of significant associations shared the same cis-eQTLs which are associated with the gene in the neighbor of target genes identified from GBM, 52 (40%) of these associations are classified as Category 1. Many eQTLs have been linked to gene expression in multiple cell lines and normal tissues from different studies. The shared cis-eQTL associations between GBM and cell lines/normal tissues in RegulomeDB are listed in Table S4.

Using *GSTT1* gene as an example we can examine in detail the sharing of cis-eQTLs between GBM and cell lines/normal tissues. As shown in Table S5, 27 out of 28 cis-eQTLs from this study have previously been linked to the expression of *GSTT1* in one or more normal tissues/cell lines including lymphoblastoid, monocytes, t-cells, and cortex. Thirteen of them were classified as Category 1 in RegulomeDB.

### Cis-eQTL associations shared between GBM samples and Breast Cancer samples

Having discovered that there is a great sharing of cis-eQTL associations between GBM samples and the normal tissues/cell lines, we next investigated the sharing of cis-eQTL associations between different cancers and different published studies by comparing the cis-eQTL associations identified from our study in GBM with those from a published Breast Cancer eQTL study [7], as well as a recently published GBM eQTL study [9]. Of 985 significant cis-eQTL associations, 242 (25%) unique associations are common to those identified from the Breast Cancer study [7] (Figure 1B & Table S6), with 241 common to those from ER-Positive Breast Cancer and 22 common to those from ER-Negative Breast Cancer. However, there is no overlap between the cis-eQTL associations of GBM identified from our study and those from the published study [9].

### Correlation of eQTL genes with patient outcome

**Figure 2:**
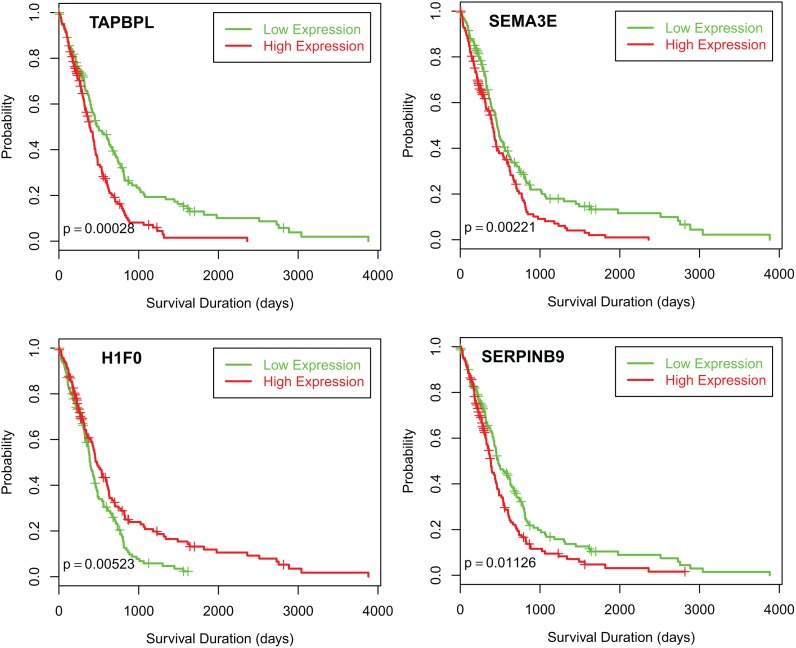
Kaplan-Meier survival plots for cis-eQTL target genes TAPBPL, SEMA3E, H1F0 and SERPINB9. The overall survival of GBM patients was used for the survival analysis. Expression values of a gene were dichotomized into high and low expression using the median as a cutoff. Green line: low expression and red line: high expression.

We are interested in determining whether the expression of any eQTL genes is associated with aggressive clinical behavior. Survival analysis using Cox proportional hazards model and logrank test showed that the expressions of 13 eQTL genes (*TAPBPL*, *SEMA3E*, *H1F0*, *SERPINB9*, *HMBOX1*, *RCAN1*, *NAA38*, *MGST3*, *TMBIM4*, *JUNB*, *THNSL2*, *IL4R*, and *LY75*) are significantly associated with patient overall survival (Table 1). The Kaplan-Meier plots for the first 4 genes are presented in Figure 2, the rest of them are shown in Figure S3.

**Table 1:** cis-eQTL target genes with significant association with patient survival.

GENE	GENEID	CHR	CYTOBAND	cis-eQTL COUNT	logrank. Pvalue	HR	95% CI
TAPBPL	55080	12	12p13.31	8	0.00031	1.648	1.256–2.163
SEMA3E	9723	7	7q21.11	4	0.00237	1.512	1.158–1.973
H1F0	3005	22	22q13.1	13	0.00537	0.682	0.52–0.893
SERPINB9	5272	6	6p25	3	0.01169	1.405	1.079–1.83
HMBOX1	79618	8	8p21.1	13	0.01416	0.719	0.552–0.937
RCAN1	1827	21	21q22.12	4	0.01478	1.389	1.067–1.809
NAA38	51691	7	7q31.1-q31.3	1	0.02694	0.743	0.571–0.967
MGST3	4259	1	1q23	2	0.03041	0.748	0.574–0.973
TMBIM4	51643	12	12q14.1-q15	15	0.03191	1.337	1.026–1.743
JUNB	3726	19	19p13.2	1	0.03537	1.328	1.019–1.729
THNSL2	55258	2	2p11.2	14	0.04171	1.318	1.01–1.719
IL4R	3566	16	16p12.1-p11.2	1	0.0446	1.313	1.007–1.712
LY75	4065	2	2q24	2	0.04755	1.304	1.003–1.696

Using gene expression data of 10 organ-specific control samples for GBM available from TCGA, we were able to investigate whether these target genes are differentially expressed in GBM tumor samples. Eleven out of thirteen genes (*TAPBPL*, *H1F0*, *SERPINB9*, *HMBOX1*, *RCAN1*, *NAA38*, *MGST3*, *TMBIM4*, *JUNB*, *IL4R*, and *LY75*) have significant differential expressions in tumor and control (p<0.05, Table S7). The expression of *TAPBPL*, *SERPINB9*, *RCAN1*, *TMBIM4*, *JUNB*, *IL4R*, and *LY75* is significantly up-regulated in tumor samples with their high expression associated with a poor outcome; the expression of *MGST3* is down-regulated in tumor samples with its low expression associated with poor prognosis (Figure 2, Figure S3 and Table S7).

**Figure 3:**
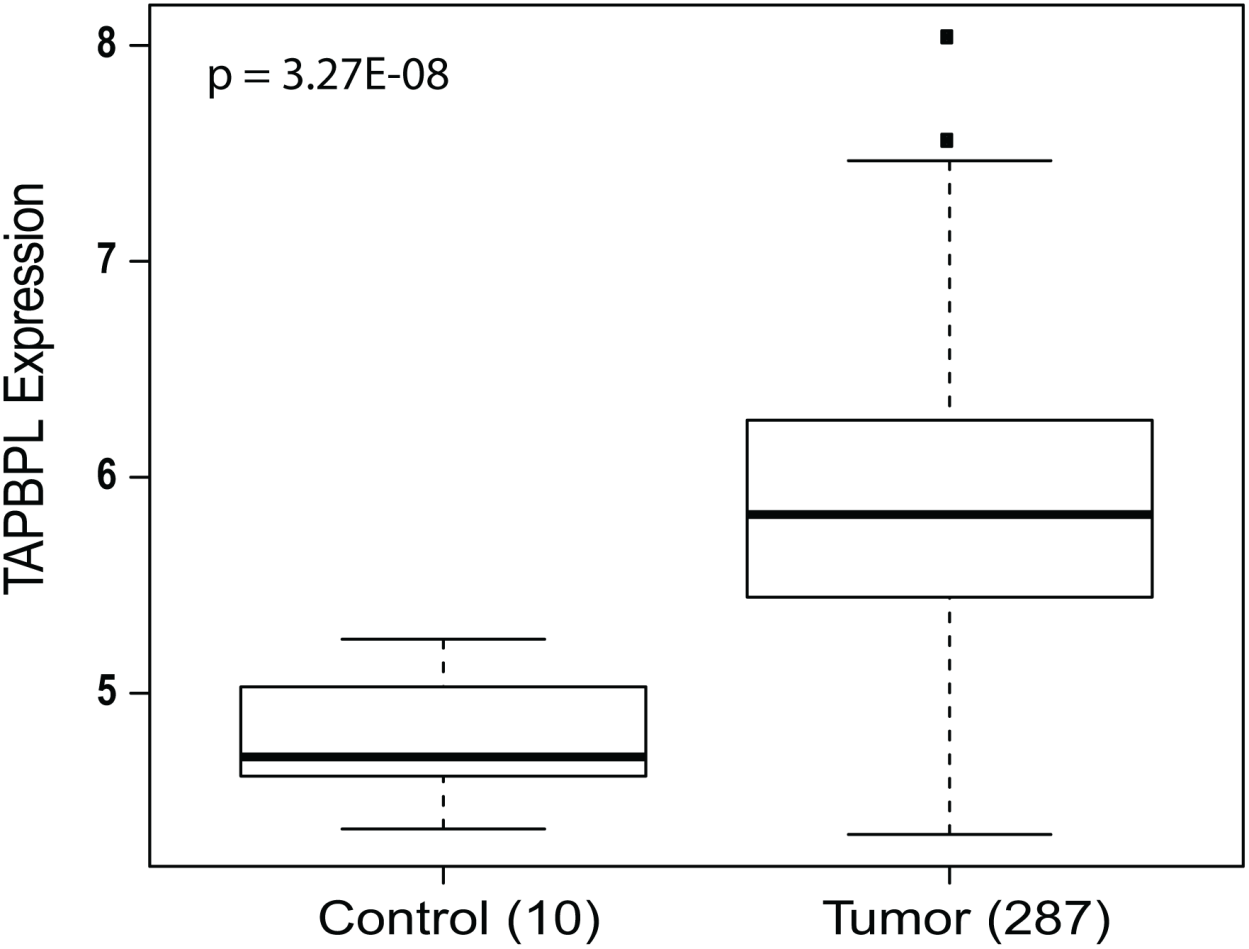
TAPBPL expression in GBM tumors and organ-specific controls. TAPBPL expression was significant higher in GBM tumor samples compared to organ-specific control samples (p = 3.27E-08).

**Figure 4:**
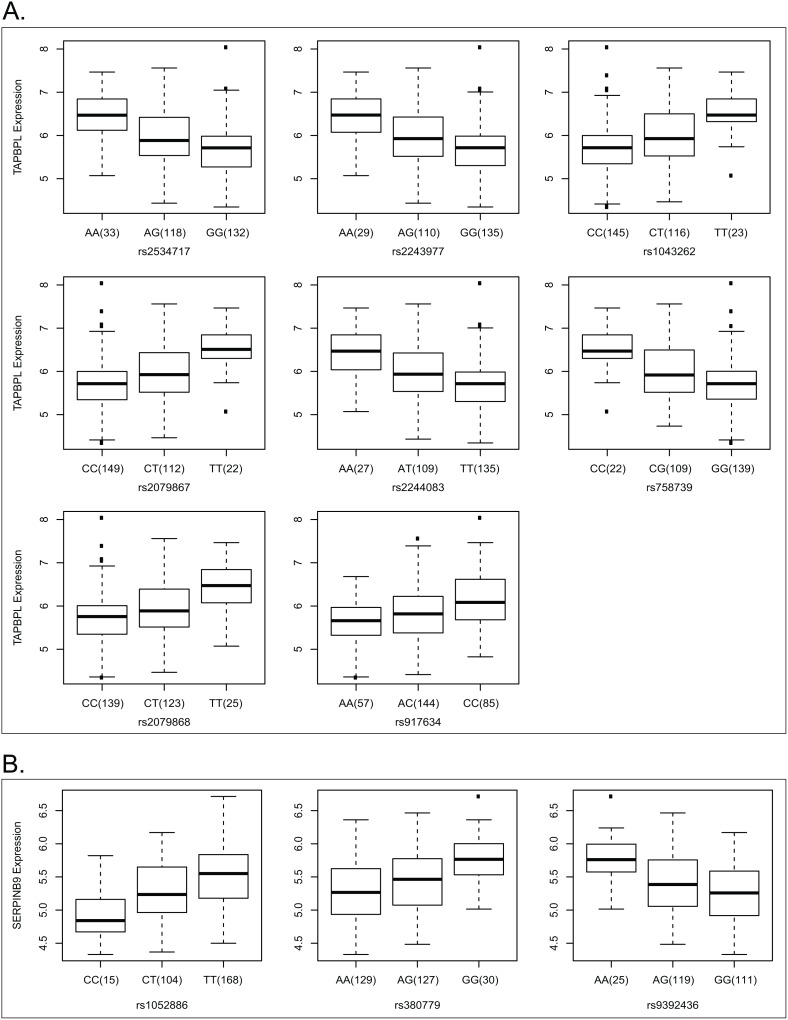
Correlation of gene expression and genotypes. **A.** Correlation between TAPBPL gene expression and different genotype groups of cis-eQTLs in GBM samples. Eight cis-eQTLs were significantly associated with TAPBPL expression. All of these cis-eQTLs have been reported to be shared between GBM tumors and monocytes/lymphoblastoid. **B.** Correlation between SERPINB9 gene expression and different genotype groups of cis-eQTLs in GBM samples. Three cis-eQTLs were significantly associated with SERPINB9 expression with cis-eQTL rs380779 be reported to beshared between GBM tumors and monocytes; and rs1052886 shared between GBM tumors and monocytes/T-cells.

There exists a strong association between *TAPBPL* expression and GBM patient survival (p<0.001). A higher expression of *TAPBPL* tends to be associated with a poor outcome. A significant increase of *TAPBPL* expression level was observed in tumor samples when compared to the organ-specific control samples (Figure 3, p<0.001). We identified 8 significant cis-eQTLs linked to the expression of *TAPBPL* (Table 1); the association between *TAPBPL* expression and genotype of each cis-acting SNP was shown in boxplots (Figure 4A). All cis-eQTLs of *TAPBPL* gene have been previously identified as cis-eQTLs in human monocytes and some also in lymphoblastoid cell lines [13]–[17]; 3 of them (rs2243977, rs2244083, and rs2534717) were classified as Category 1 in RegulomeDB and likely to affect binding to the target gene [12] (Table 2). All eight cis-eQTL/*TAPBPL* associations are represented in Breast Cancer [7] (Table S6).

**Table 2:** cis-eQTL/TAPBPL associations shared between GBM and cell lines in Regulome DB.

RSid	Chr	Pos	Gene	RegulomeDB_score	eQTL_status	cell lines/tissues	Reference
rs2243977	chr12	6569982	TAPBPL	1f	cis eQTL	Monocytes, Lymphoblastoid	12–16
rs2244083	chr12	6570538	TAPBPL	1b	cis eQTL	Monocytes, Lymphoblastoid	12–14, 16
rs2534717	chr12	6573748	TAPBPL	1d	cis eQTL	Monocytes, Lymphoblastoid	12–13, 16
rs2079867	chr12	6622110	TAPBPL	6	cis eQTL	Monocytes, Lymphoblastoid	12–13, 16
rs2079868	chr12	6622345	TAPBPL	6	cis eQTL	Monocytes	12
rs758739	chr12	6626368	TAPBPL	6	cis eQTL	Monocytes, Lymphoblastoid	12–13
rs917634	chr12	6631168	TAPBPL	6	cis eQTL	Monocytes	12
rs1043262	chr12	6639087	TAPBPL	6	cis eQTL	Monocytes	12

Note: These 8 significant cis-eQTL/TAPBPL associations are also common to those identified from Breast Cancer^7^ (Table S6).

For the rest 12 target genes associated with GBM patient survival, the number of cis-eQTLs linked to the expression of target gene varies. Genes *H1F0*, *HMBOX1*, *TMBIM4*, and *THNSL2* had a relatively higher number of significantly associated eQTLs ranging from 13 to 14. All cis-eQTLs linked to the expression of *H1F0*, *HMBOX1*, and *THNSL2* genes have been previously reported as cis-eQTLs in one or multiple cell lines and normal tissues including monocytes and lymphoblastoid cell lines, cortex, pons, and cerebellum. Some of these cis-eQTLs (8 for *H1F0*, 6 for *HMBOX1*, and 4 for *THNSL2*) were classified as Category 1 in RegulomeDB. The cis-eQTL associations of *H1F0*, *HMBOX1*, and *THNSL2* genes are also represented in Breast Cancer (Table S6). However, only 3 out of 14 eQTLs linked to *TMBIM4* expression have been reported previously as cis-eQTLs with two of them classified as Category 1 in RegulomeDB. Of those target genes with fewer associated cis-eQTLs (ranging from 1 to 4), the cis-eQTL sharing between GBM and cell lines/normal tissues seemed less. Three SNP loci were linked to the expression of *SERPINB* (Figure 4B) with 2 having been reported in T-cells and/or monocytes^12,17^ and classified as Category 1; both eQTLs linked to the expression of *MGST3* have been reported in lymphoblastoid and monocytes^12–14^ and classified as Category 1. However, there was no evidence available in RegulomeDB for cis-eQTLs linked to the expression of the rest six genes (*SEMA3E*, *RCAN1*, *NAA38*, *JUNB*, *IL4R*, and *LY75*). The boxplot presentation of cis-eQTL association for *TMBIM4* was also shown in Figure S4.

## Discussion

In this study, we have utilized a large collection of GBM data sets obtained from The Cancer Genome Atlas TCGA to systematically investigate germline contribution to tumor gene expression in GBM. Approximately 57% of the significant cis-eQTL associations identified in GBM from this study have been previously reported either in cell lines or normal tissues based on the information from RegulomeDB. RegulomeDB includes high-throughput, experimental data sets from ENCODE and other sources, as well as computational predictions and manual annotations to identify putative regulatory potential and identify functional variants [12]. About 43% of the shared cis-eQTL associations between GBM and cell lines/normal tissues also have known functional annotation of likely significance [12]. Previous reports suggested 20–30% overlap of eQTLs between different tissues [7], [18]–[19]. The high overlap of cis-eQTL findings between the current GBM study and the previous normal tissue and cell line studies provides us the confidence of detecting true positive cis-eQTLs and it also suggests that the same set of genetic variations existing in normal tissues are making contributions to tumor gene expression and they might be involved in cancer pathogenesis.

Previous studies have applied eQTL analysis approach in GBM samples for discovering the possible causal pathways [20]–[21]. A recent study has also applied eQTL analysis approach to systematically identify eQTLs in TCGA GBM samples [9]. Surprisingly, the significant cis-eQTL associations identified from this report and our study have no overlap. We noticed a few methodology differences between two studies. First, different subsets of GBM samples were used. Second, different levels of gene expression data were used. Third, different filtering methods were used. Fourth, different gene expression values were used. Specifically, the Shapk study used fold change in expression level compared to 10 control samples rather than raw expression levels in the tumor, which we used. All of these methodology differences may lead to non-overlapping results. In addition, the Shapk study has compared their cis-eQTL associations with those identified from the Breast Cancer eQTL study [7] and found only a single common association. Different from their observations, we have found that 25% (n = 242) of the significant cis-eQTL associations of GBM from our study are represented in those indentified from the Breast Cancer [7]. Although we applied different eQTL analysis approaches and different FDR cutoffs on different cancer types (Breast Cancer vs. GBM), we have reached a reasonably high overlapping rate (25%), suggesting that the common cis-eQTL associations highly exist across cancers as well.

GWAS has identified a few susceptibility variants in glioblastoma or glioma; 12 loci at the regions of 20q13.33 (*RTEL1*), 5p15.33 (*TERT*), 9p21.3 (*CDKN2BAS*), 7p11.2 (*EGFR*), 8q24.21 (*CCDC26*), and 11q23.3 (*PHLDB1*) [22]–[23] have been collected in the NHGRI GWAS catalog (http://www.genome.gov/gwastudies/). None of the cis-eQTLs identified from this study is among these 12 loci. We checked the data set and found that the filtered genotype SNP6 data doesn’t contain SNPs at the region of *CDKN2BAS* and *CCDC26* genes and the TCGA level 3 gene expression data doesn’t contain *PHLDB1*, *RTEL1* and *TERT* genes. Only the region of EGFR gene contains both genotype data and gene expression data, but none of the cis-eQTL associations was significant. There are a few target genes (*GSTM3*, *GSTT1*, *MGMT*, *MTR*, and *IL4R*), for which their polymorphisms have been reported to be associated with brain tumors based on the Genetic Association Database (http://geneticassociationdb.nih.gov), which are not reported in the GWAS catalog. The Genetic Association Database [24] is an archive of all human genetic association studies of complex diseases and disorders. As such, it includes summary data extracted from published papers in peer reviewed journals on candidate gene and GWAS studies. For example *GSTT1* and *GSTM3* belong to a family of glutathione S-transferases (GSTs), which are important enzymes involved in detoxification of varieties of environmental carcinogens. The association between GST variants and risk of glioma has been extensively studied, especially for GSTT1 polymorphisms due to its null deletion genotypes that have no enzymatic activity. However the association results remained inconsistent [25]–[27].

We identified thirteen cis-eQTL target genes (*TAPBPL*, *SEMA3E*, *H1F0*, *SERPINB9*, *HMBOX1*, *RCAN1*, *NAA38*, *MGST3*, *TMBIM4*, *JUNB*, *THNSL2*, *IL4R*, and *LY75*) with their expression associated with GBM survival. The cis-eQTL associations of 7 genes (*TAPBPL*, *H1F0*, *SERPINB9*, *HMBOX1*, *MGST3*, *TMBIM4*, *THNSL2*) are shared between GBM and cell lines/normal tissues; the association of 4 genes (*TAPBPL*, *H1F0*, *HMBOX1*, *THNSL2*) are common between GBM and Breast Cancer. Several of these genes have functions known to be important in cancer biology. *TAPBPL* is reported to be a component of the major antigen histocompatibility complex (MHC) class I presentation pathway and the expression of *TAPBPL* decreases the rate of MHC class I maturation [28]. MHC class I down-regulation is a general mechanism by which tumor cells can escape from T-cell-mediated immunity. *SERPINB9* is a human proteinase inhibitor expressed in certain normal cell types and cancer cells of different origin and can protect the cancer cells from granzyme B (GrB)-mediated apoptosis [29]. High level of *SERPINB9* expression can block not only GrB/perforin-mediated death pathway but also the FasL/Fas death pathway [30]. *HMBOX1*, homeobox telomere binding protein 1, is a novel transcription factor. A recent study identified *HMBOX1* as a factor that binds to both telomeres and telomerase to bring them in close proximity, and positively regulates telomere length [31]. Telomeres are implicated in genome integrity control and carcinogenesis. Cancer cells depend on telomere maintenance mechanisms in order to gain immortal proliferation capacity and to prevent genetic chaos induced by telomere dysfunction [32]. The finding that *HMBOX1* supports telomerase-dependent telomere elongation might indicate the possible critical role of *HMBOX1* in cancer pathogenesis. *TMBIM4*, transmembrane Bax inhibitor–containing motif protein 4, is also known as Golgi antiapoptic protein (*GAAP*). GAAPs have similar lengths and hydrophobicity profiles, and they have important and evolutionarily conserved functions. Human *GAAP* (*hGAAP*) has been reported to inhibit apoptosis and promote cell adhesion and migration via the stimulation of store-operated Ca2+ entry and calpain 2; the up-regulation of its mRNA has been observed in some human cancers [33]–[34]. This evidence suggests that *hGAAP* might also contribute to cancer progression and metastasis.

In summary, we have demonstrated an effective way to systematically assess the genetic contribution to expression levels in tumor using publicly accessible data sets. Our study provides a global view of the germline influence on GBM gene expression and suggests a significant sharing between cis-eQTL association results from GBM and those derived from cell lines and normal tissues, as well as those from Breast Cancer. The observation of differential survival based on the expression of genes associated with eQTLs might be important to the understanding of genetic contribution to GBM cancer pathogenesis and prognosis.

## Supporting Information

Figure S1 **The cis-eQTL analysis flowchart.**
(TIF)

Figure S2 A. GSTT1/eQTL association scan in the region of chromosome 22 around GSTT1 gene. The peak harbors 28 significant cis-eQTLs associated with GSTT1. B. Haploview plot defining hapotype block structure in 1 Mb region of GSTT1 gene. SNPs shown in red are significant cis-eQTLs.
(TIF)

Figure S3 **Kaplan-Meier survival plots for cis-eQTL target genes HMBOX1, IL-4R, JUNB, NAA38, LY75, MGST3, RCAN1, THNSL2, and TMBIM4.** The overall survival of GBM patients was used for the survival analysis. Expression values of a gene were dichotomized into high and low expression using the median as a cutoff. Green line: low expression and red line: high expression.
(TIF)

Figure S4 **Correlation between TMBIM4 gene expression and dierent genotype groups of cis-eQTLs in GBM samples.** Fifteen cis-eQTLs were signi-cantly associated with TMBIM4 expression. Only 3 cis-eQTLs (rs1168765, rs1185888, and rs2870783) have been reported to be shared between GBM tumors and monocytes.
(TIF)

Table S1 (XLSX)

Table S2 (XLSX)

Table S3 (XLSX)

Table S4 (XLSX)

Table S5 (XLSX)

Table S6 (XLSX)

Table S7 (XLSX)
