# Supplementary figures and images for: Systematic Genetic Analysis Identifies Cis-eQTL Target Genes Associated with Glioblastoma Patient Survival
Source: PLoS One. 2014 Aug 18;9(8):e105393. doi: 10.1371/journal.pone.0105393 (PMC4136869; doi:10.1371/journal.pone.0105393)

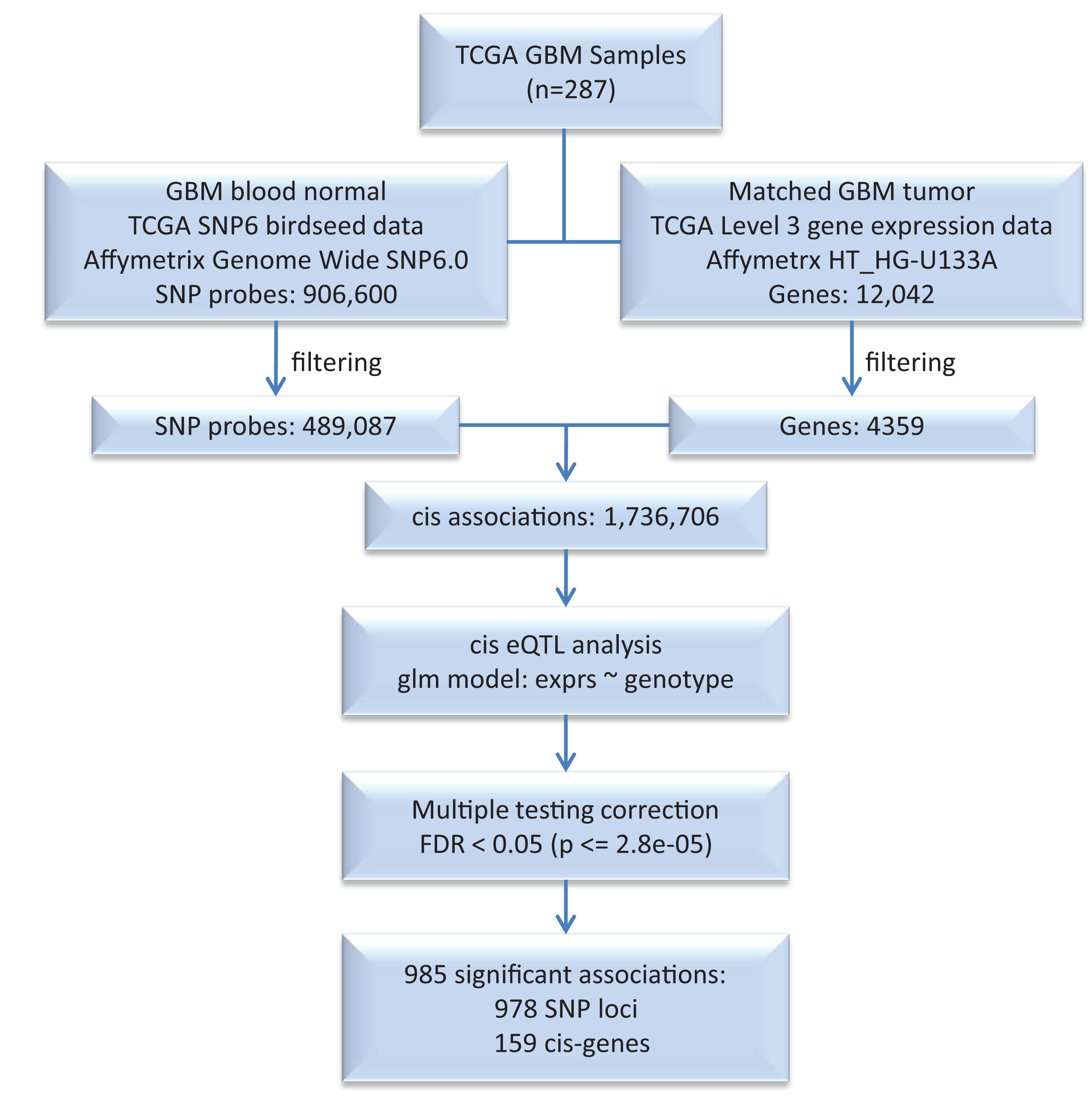

Supplement: Figure S1 — The cis-eQTL analysis flowchart. (TIF) [file pone.0105393.s001.tif]

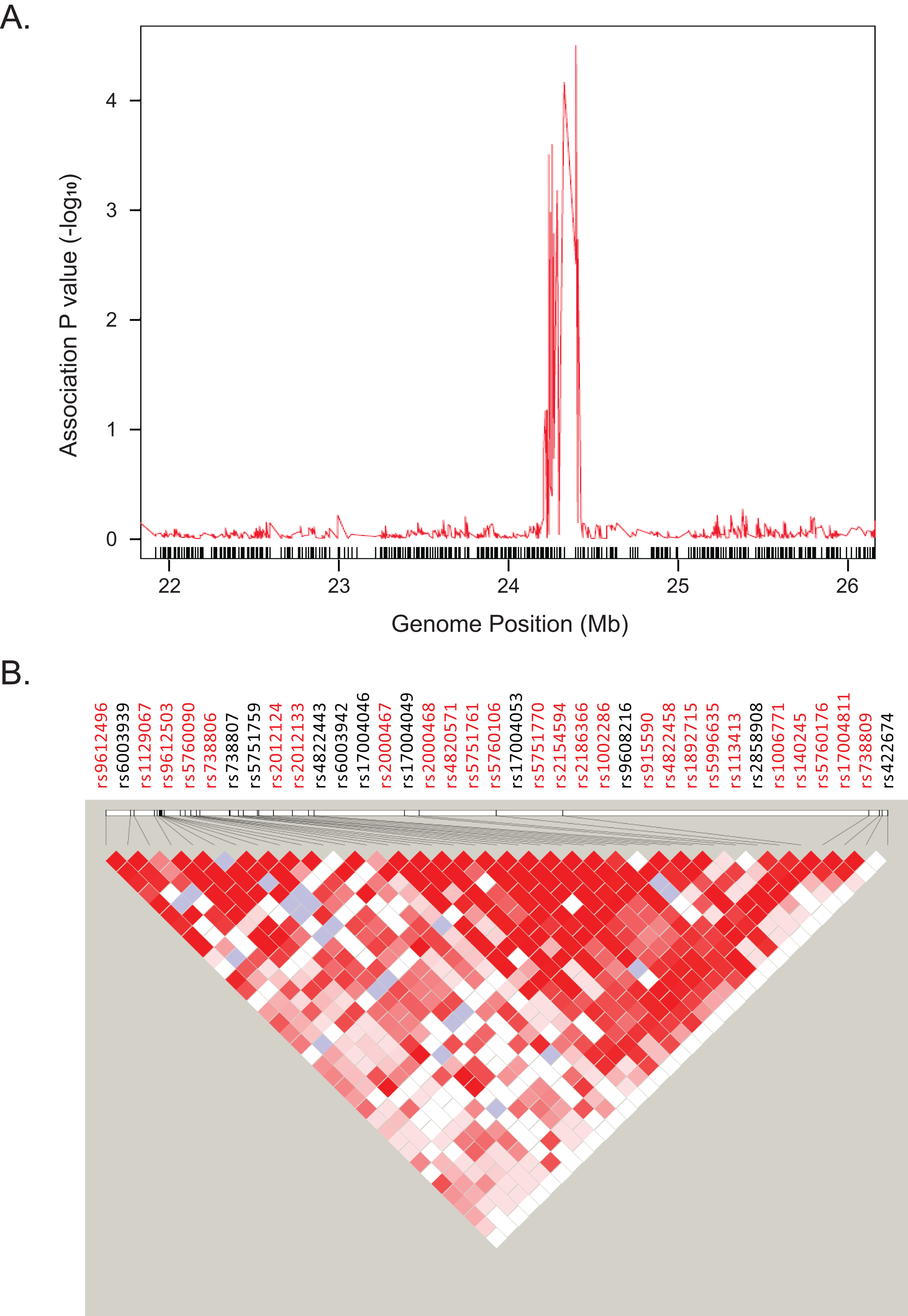

Supplement: Figure S2 — A. GSTT1/eQTL association scan in the region of chromosome 22 around GSTT1 gene. The peak harbors 28 significant cis-eQTLs associated with GSTT1. B. Haploview plot defining hapotype block structure in 1 Mb region of GSTT1 gene. SNPs shown in red are significant cis-eQTLs. (TIF) [file pone.0105393.s002.tif]

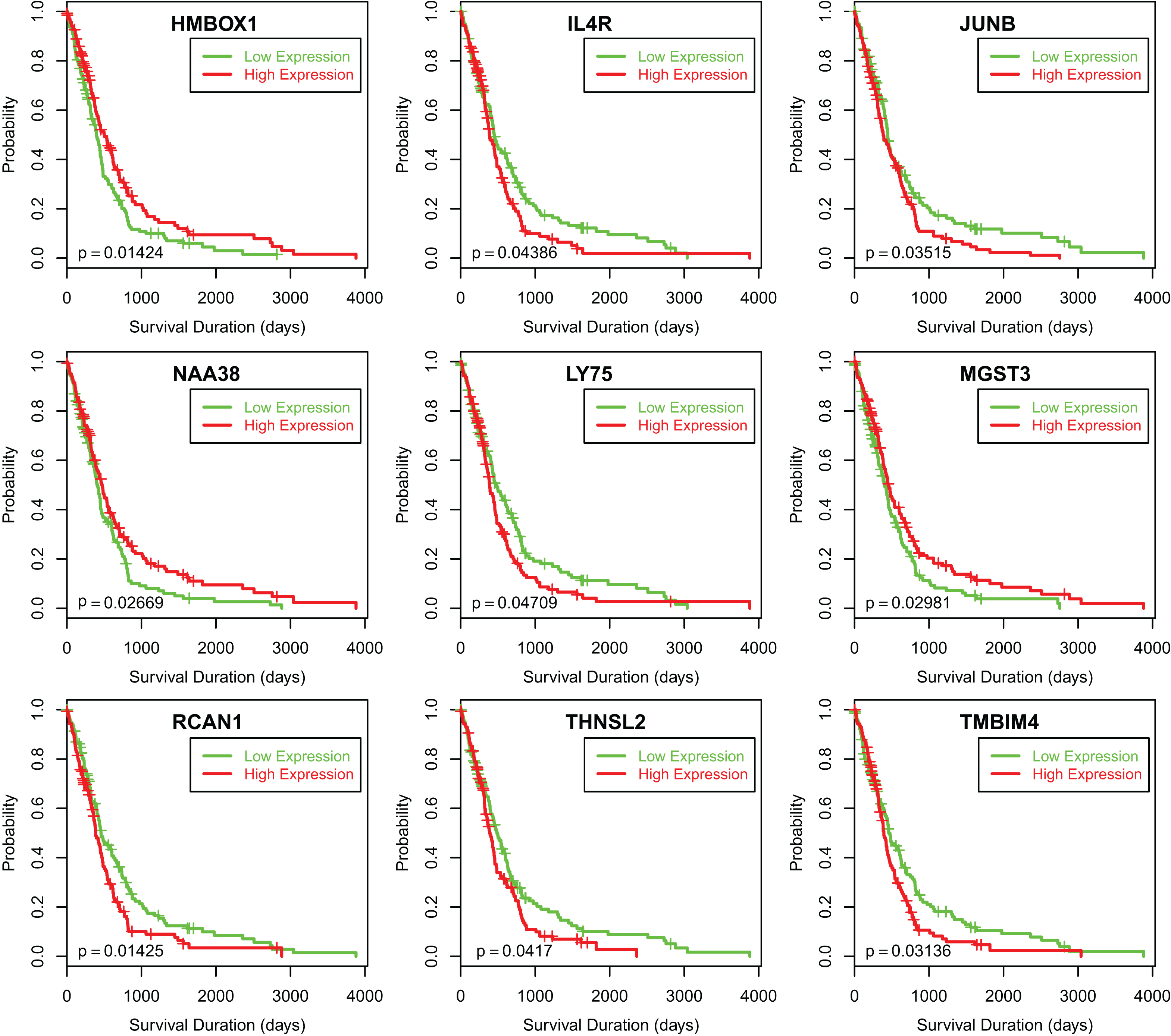

Supplement: Figure S3 — Kaplan-Meier survival plots for cis-eQTL target genes HMBOX1, IL-4R, JUNB, NAA38, LY75, MGST3, RCAN1, THNSL2, and TMBIM4. The overall survival of GBM patients was used for the survival analysis. Expression values of a gene were dichotomized into high and low expression using the median as a cutoff. Green line: low expression and red line: high expression. (TIF) [file pone.0105393.s003.tif]

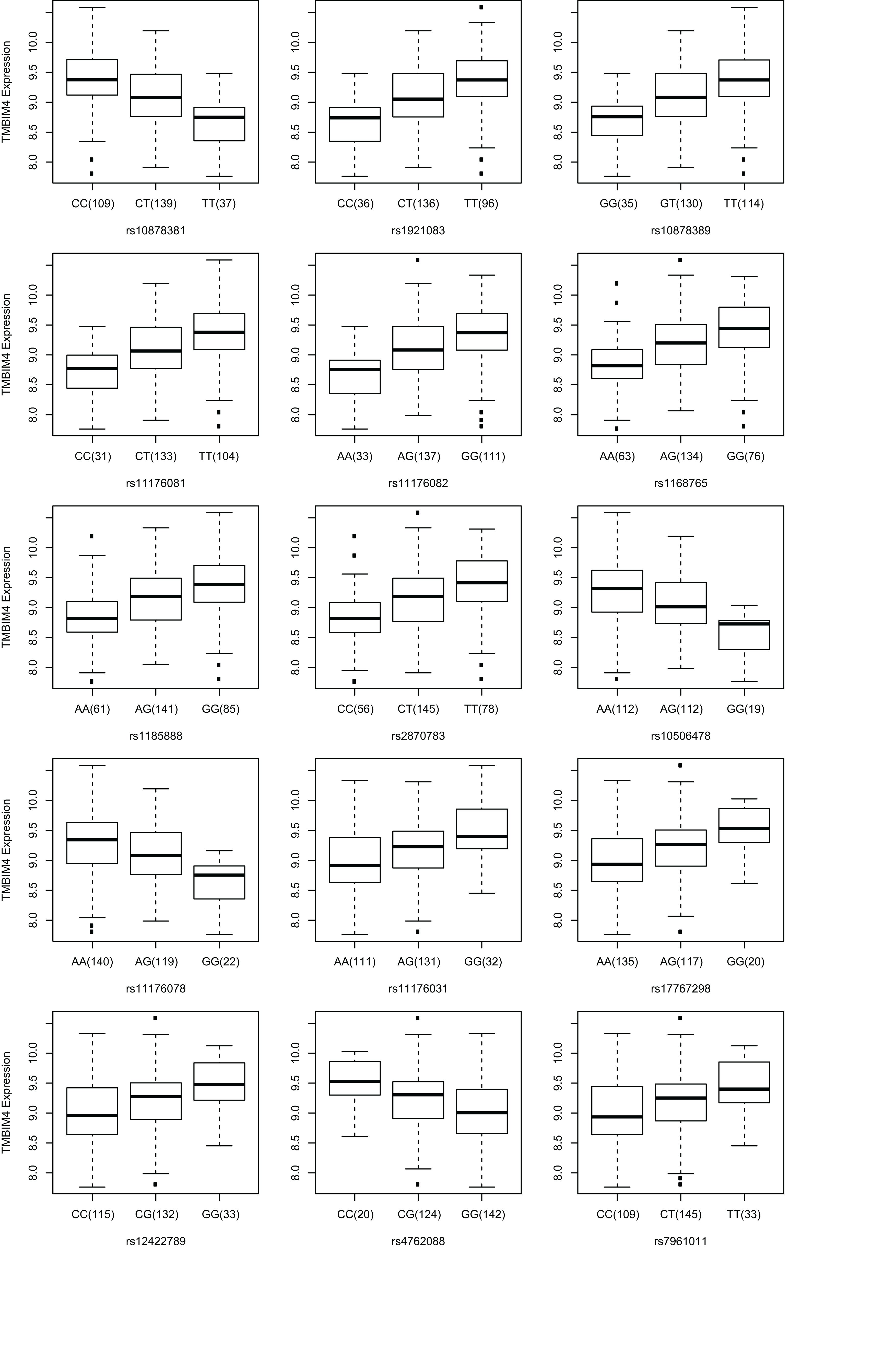

Supplement: Figure S4 — Correlation between TMBIM4 gene expression and dierent genotype groups of cis-eQTLs in GBM samples. Fifteen cis-eQTLs were signi-cantly associated with TMBIM4 expression. Only 3 cis-eQTLs (rs1168765, rs1185888, and rs2870783) have been reported to be shared between GBM tumors and monocytes. (TIF) [file pone.0105393.s004.tif]
